# An overview of randomized clinical trials of fixed‐ratio combinations of basal insulin plus GLP‐1RA (injectable therapy): Lessons for advancing therapy in people with type 2 diabetes

**DOI:** 10.1111/dom.16616

**Published:** 2025-07-17

**Authors:** Geremia B. Bolli, Francesca Porcellati, Paola Lucidi, Carmine G. Fanelli, Gianluca Perseghin, Michael Horowitz, David R. Owens

**Affiliations:** ^1^ Department of Medicine and Surgery Perugia University Medical School Perugia Italy; ^2^ Centro Diabetologia Asl 2 Umbria ‘Centro Storico’ Foligno Italy; ^3^ Department of Medicine and Surgery Università degli Studi di Milano Bicocca & Policlinico, di Monza Monza Italy; ^4^ Endocrine and Metabolic Unit, Royal Adelaide Hospital University of Adelaide Adelaide South Australia Australia; ^5^ Cardiff University Medical School Cardiff UK

**Keywords:** basal insulin, fixed‐ratio combinations basal insulin GLP‐1RAs, GLP‐1RA, type 2 diabetes

## Abstract

Advancing therapy in T2DM with injectables, i.e., basal insulin (BI) and GLP‐1 receptor agonists (GLP‐1RAs) is recommended after the failure of oral glucose lowering agents (OGLAs), BI alone, or BI in combination with OGLAs, especially in persons with, or at high risk of atherosclerotic cardiovascular disease (ASCVD). BI and GLP‐1RAs can be administered separately or as fixed‐ratio combinations (FRCs) for daily use (degludec+liraglutide, IDegLira, glargine‐100 + lixisenatide iGlarLixi) or weekly use (icodec+semaglutide, IcoSema). The currently available FRCs IDegLira and iGlarLixi differ in their respective BI as well as GLP‐1RA components. Liraglutide predominantly stimulates glucose‐dependent endogenous insulin secretion in response to nutrient challenges. In contrast, the rapid‐acting lixisenatide primarily delays gastric emptying over a few hours post‐dosing with little or no impact on insulin secretion. IDegLira in DUAL studies and iGlarLixi in LixiLan studies appear to have equivalent lowering effects on HbA1c, although IDegLira achieves a greater reduction in body weight. The weekly FRC IcoSema is superior to weekly insulin icodec (COMBINE 1), to semaglutide (COMBINE 2), and non‐inferior to basal‐bolus insulin therapy (COMBINE 3). Comparison of IcoSema with glargine‐100 is ongoing (COMBINE 4). However, all FRCs are limited by the low GLP‐1RA dose relative to the insulin delivered. Whenever higher GLP‐1RA doses are required (i.e., in obese people), the option of separate dosing of BI and GLP‐1RA with independent titration of each component should be considered.

## 1 INTRODUCTION

In individuals with recently diagnosed type 2 diabetes mellitus (T2DM), effective control of hyperglycaemia reduces the risk of long‐term vascular complications, primarily microvascular in the short term,^1^, ^2^ and macro‐vascular over a more prolonged period of time stage.^3^, ^4^, ^5^

Given the progressive nature of T2DM,^6^ it is often necessary to advance therapy with injectable glucose lowering agents, either basal insulin (BI) or GLP‐1RA, or in combination whenever previous therapy has failed to bring HbA1c level to target.^7^ The rationale for the co‐administration of BI and GLP‐1RA relies on their different and complementary mechanisms of action, since BI primarily lowers fasting plasma glucose (FPG) while GLP‐1RAs reduce post‐prandial plasma glucose (PPG). Thus, the combination of these two classes of drugs enhances glycaemic control, reduces body weight whilst limiting the risk of hypoglycaemia (Table 1).^8^ It may also provide cardio‐renal protection in people with established complications.^7^

**TABLE 1:** Complementary mechanisms of action and add‐on glucose lowering effects of the association of basal insulin (BI) and GLP‐1RAs (modified and reproduced, with permission of the American Diabetes Association).^8^

	Basal insulin	GLP‐1RAs	Basal insulin+GLP‐1RAs
Effects on:			
FPG	↓ ↓ ↓	↓	↓ ↓ ↓ ↓
PP‐PG	↓	↓ ↓ ↓	↓ ↓ ↓ ↓
HbA1c	↓ ↓ ↓	↓ ↓	↓ ↓ ↓ ↓ ↓
Hypoglycaemia	↑	←→	← → or ↑
BW	↑	↓ ↓	← → or ↓
Hunger	←→	↓ ↓ ↓	↓ ↓ ↓

Combined formulations of BI and GLP‐1RA became available nearly a decade ago. Fixed‐ratio Combinations (FRCs) have since become available, providing a simplified titration and dosing option, with only one rather than two separate daily or weekly injections of the two components. However, in the largely heterogeneous population with T2DM with different requirements of BI relative to GLP‐1RA, this convenience of FRCs must be balanced against the limitation in titration flexibility.

The article aims, first, to review the evidence for the efficacy and safety of FRCs currently available and those to come in individuals with T2DM from RCTs and secondary analyses, and secondly to explore how these studies help to identify which individuals are most likely to benefit from FRCs when advancement of therapy is needed.

## 2 THE FIXED RATIO COMBINATIONS BI+GLP‐1RA AVAILABLE FOR CLINICAL USE

At present there are two FRCs available on the market for daily use in type 2 diabetes: degludec+liraglutide (IDegLira)^9^ approved by EMA in 2014 and FDA in 2016, and glargine‐100 + lixisenatide (iGlarLixi)^10^ approved by FDA in 2016 and EMA in 2017. An additional FRC for weekly use, icodec plus semaglutide (IcoSema) is under development and not yet approved.^11^, ^12^, ^13^

### 2.1 IDegLira

The complementary effects of degludec and liraglutide have been demonstrated in two studies where liraglutide was either added on to degludec^14^ or degludec added to liraglutide^15^ in people not at the target HbA1c with either degludec or liraglutide alone. Each 100 units of degludec of IDegLira contain 3.6 mg liraglutide, i.e., each unit dose of degludec delivered by a pre‐filled pen delivers 0.036 mg of liraglutide.^9^ However, even assuming that the “total” plasma concentration of degludec and liraglutide (both acylated products reversibly bound to albumin) is related to its “free” concentrations and metabolic activities, the exposure to liraglutide is 11% lower when the co‐formulation is compared with separate administration of degludec and liraglutide.^16^

### 2.2 iGlarLixi

Glargine (100 U/mL)^17^ and the GLP‐1RA lixisenatide^18^, ^19^ have similar physico‐chemical features, allowing stable mixtures at ratios of 100/33 and 100/50 respectively.^10^ The two different pre‐filled pens of iGlaLixi ratio deliver from 30 up to 60 units of IGla‐100 along with 10–20 μg of lixisenatide, or 10 to 40 units of iGla‐100 with 5–20 μg of lixisenatide, respectively. Another pre‐filled pen of iGlaLixi ratio (5–20 units of iGla‐100 with 5–20 μg of lixisenatide) is available in other countries, including Japan. The complementary effects of lixisenatide and IGla‐100 given separately were demonstrated in the GetGoal‐L and GetGoal Duo‐1 trials, showing superior effects on PPG and HbA1c compared with BI alone without weight gain or increased hypoglycaemia risk.^20^, ^21^ In healthy participants and people with T1DM and T2DM, the pharmacokinetic (PK) profile of iGlarLixi vs. IGla‐100 and lixisenatide given separately showed similar profiles for IGla‐100; however, with a 22%–34% decrease in maximum plasma concentration of lixisenatide with the FRC.^22^, ^23^

### 2.3 IcoSema

The most recent FRC of BI and GLP‐1RA is the once‐aa‐week co‐formulation of the weekly BI icodec^24^ plus the weekly GLP‐1RA analogue semaglutide^25^ (IcoSema).^11^, ^12^, ^13^, ^26^ Each unit dose of icodec delivers 0.0029 mg of semaglutide with a maximum weekly dose corresponding to 350 U of icodec (equivalent to 50 U per day) and 1.0 mg of semaglutide (0.14 mg/day). The PK of semaglutide injected alone or as FRC with icodec in IcoSema differs substantially, with the s.c. absorption of semaglutide much faster when injected as IcoSema.^26^ This surprising finding is considered to reflect competition at the s.c. injection site, as both components are acylated and, with icodec more avidly bound to albumin, less albumin remains available for semaglutide, which is, therefore, more easily absorbed into circulation.^26^

## 3 THE RCTs OF FRCs FOR ONCE‐DAILY DOSING

All clinical trials run for the development and approval of the FRCs IDegLira and iGlarLixi by the agencies have compared the FRCs versus only one of the two components, either BI or GLP‐1RA, or less frequently versus both the two components; however, versus only one at each time. Thus, no study has compared the FRCs versus both the two components given together as separate dosing and independently titrated. The studies have tested the FRCs in people with T2DM failing to reach the target HbA1c during therapy with oral glucose lowering agents (OGLA), and also in populations with longer diabetes duration on BI treatment with/without OGLA, or on GLP‐1RA, and failing to reach the target HbA1c.

### 3.1 The studies with IDegLira (DUAL I‐IX, and DUAL high)

The FRC iDegLira has been studied in the ‘DUAL’ clinical trial programme which includes in total 10 studies (Table 2).

**TABLE 2:** Randomized controlled trials with the once‐daily FRC iDegLira (DUAL). The values of age, BMI, diabetes duration and HbA1c are intended as means. The outcomes are expressed as effect of iDegLira vs. comparator.

STUDY	Treatment duration (weeks)	Baseline treatment	Comparator(s)	Number of subjects	Age (years)	BMI (kg/m^2^)	Diabetes Duration (years)	HbA1c (%)	Outcomes
DUAL I^27^	26	Metformin ± pioglitazone	iDeg liraglutide	1.663	55	31.2	6.9	8.3	HbA1c ↓ vs. both Deg and Lira
DUAL II^28^	26	BI + met ± SU	IDeg	413	58	33.7	10.5	8.8	HbA1c ↓ BW ↓ Hypogl. =
DUAL III^29^	26	GLP‐1RA ± OGLA^a^	GLP‐1RA	438	58	33	10.4	7.75	HbA1c ↓ BW ↓ Hypogl. ↑
DUAL IV^30^	26	OGLA^a^	Placebo	436	59.5	31.5	9.2	7.9	HbA1c ↓ BW ↑ Hypogl. ↑
DUAL V^31^	26	IGla‐100 + metformin	IGla‐100	557	58.8	31.7	11.5	8.3	HbA1c ↓ BW ↓ Hypogl. ↓
DUAL VI^32^	32	Metformin ± pioglitazone	IDegLira titration once weekly	420	57	32.5	7.3	8.2	HbA1c = BW = Hypogly. =
DUAL VII^33^	26	IGla‐100 + metformin	Basal‐bolus insulin	506	58.3	31.7	13.2	8.2	↓HbA1c = BW. ↓ Hypoglyc. ↓
DUAL VIII^34^	104	OGLA^a^	IGla‐100	1.012	56.6	32	10.1	8.5	Durability^b^↑
DUAL IX^35^	26	SGLT2 ± OGLA^a^	IGla‐100	420	56.7	31.2	9.6	8.3	HbA1c ↓ BW ↓ Hypoglyc. ↓
DUAL High^36^	26	BI ± OGLA^a^	Basal‐bolus insulin	145	54.2	32	86% of subjects <20 years^c^	10.75	↓HbA1c = BW. ↓ Hypoglyc. ↓

^a^ Oral glucose‐lowering agents.

^b^ Expressed as longer time to need for treatment intensification.^34^

^c^ Absolute diabetes duration not given.^36^

In DUAL I people with uncontrolled T2DM on metformin ± pioglitazone, naïve to injectables, were randomized to treatment with either IDegLira or IDeg or liraglutide.^27^ At the end of study, the decrease in HbA1c and the percentage of subjects at target HbA1c <7.0% were greater with IDegLira than with either of the two individual components. IDegLira lowered the body weight and risk of hypoglycaemia compared with IDeg, but less so than with liraglutide.

In DUAL II, to specifically address the contribution of liraglutide to the effects of the FRC IDegLira, people with uncontrolled T2DM on BI and metformin ± sulphonylurea were randomized to either IDegLira or IDeg with insulin dose capped at 50 units.^28^ The important contribution of liraglutide was confirmed by the superior HbA1c decrease with the FRC with lower body weight and no difference in the risk of hypoglycaemia.^28^

In DUAL III the efficacy and safety of IDegLira was compared with that of unchanged GLP‐1 receptor agonist (liraglutide or exenatide) therapy.^29^ IDegLira was superior to GLP‐1RA alone in lowering HbA1c to target, although body weight increased along with the risk of hypoglycaemia, primarily in people on sulphonylurea treatment.^29^

DUAL IV compared the add‐on of IDegLira vs. placebo in insulin‐naïve people with T2DM not at the target on sulphonylurea ± metformin.^30^ IDegLira was superior in lowering HbA1c, although both body weight and the risk of hypoglycaemia increased.^30^

DUAL V compared the efficacy and safety of IDegLira with that of continued IGlar U100 up‐titration.^31^ IDegLira was superior in lowering HbA1c primarily due to lower day‐long PPG with the advantage of lower body weight and a lower risk of hypoglycaemia.^31^

DUAL VI compared two titration algorithms, once‐ versus twice‐weekly, for IDegLira in insulin‐naïve people with T2DM not at the target with OGLA.^32^ The once‐weekly titration algorithm was non‐inferior to the twice‐weekly algorithm for HbA1c control with similar effects on body weight and risk for hypoglycaemia.^32^

DUAL VII compared the efficacy and safety of IDegLira with that of basal‐bolus insulin therapy in people with T2DM not at the target HbA1c on 20–50 units/day of IGla‐100 and metformin.^34^ IDegLira was non‐inferior to basal‐bolus insulin in lowering HbA1c, with the added benefits of less weight gain and a lower risk of hypoglycaemia.^33^

DUAL VIII assessed the durability of IDegLira as compared with IGla‐100 in people with T2DM on OGLA who needed treatment intensification.^34^ Over the 2 years of the study, IDegLira was more durable than IGla‐100 in maintaining the HbA1c target with the advantages of lower body weight and lower risk for hypoglycaemia.^34^

DUAL IX compared IDegLira versus IGla‐100 in insulin‐naïve people with T2DM not at the target HbA1c on SGLT2i ± other OGLA. IDegLira was superior in lowering HbA1c, with a greater reduction in body weight and lower risk for hypoglycaemia.^35^

The more recent DUAL High trial has compared treatment with IDegLira versus basal‐bolus insulin in people with T2DM in poor control on OGLA ± BI with HbA1c ≥9%–15%.^36^ After 26 weeks, IDegLira was non‐inferior to basal‐bolus insulin in lowering HbA1c. Body weight and risk for hypoglycaemia were also less with IDegLira.

All DUAL studies have shown more frequent adverse gastrointestinal (GI) effects (predominantly in the initial weeks of treatment followed by progressive waning in the subsequent weeks) with IDegLira as compared with OGLA, BI, and basal‐bolus insulin regimens.

#### 3.1.1 Comments to the IDegLira studies

The several DUAL studies have explored a variety of clinical circumstances in which advancing therapy with the combination of the two injectables, the BI degludec and the long‐acting GLP‐1RA liraglutide, is potentially useful in people with long diabetes duration and disease progression. Overall, several DUAL studies have demonstrated the superiority of the FRC IDegLira compared with either alone in terms of lowering of HbA1c with the added benefits of lower body weight and lower risk of hypoglycaemia. However, the more robust effect on HbA1c of the FRC containing two as compared with only one glucose‐lowering drug, on the one hand, is well expected and the other confirms the additive effects of BI and GLP‐1RA.^7^ What the DUAL studies have not demonstrated is how much the FRC IDegLira can reproduce the effects of the free combination of separate dosing of both the two components, BI degludec and GLP‐1RA liraglutide, injected and titrated separately. There are also limitations in the design of some of the DUAL studies. For example, in the DUAL II study versus BI, only people on a daily BI dose of 20–40 U (±10%) were recruited^28^ and in DUAL V^31^ the maximum dose step of IDegLira was set at 50 U. The limitation of the titration of the FRC IDegLira is suggested by DUAL I, where FPG was at a similar target with IDegLira or IDeg alone, indicating appropriate titration of the IDeg component of the FRC as compared with free dosing of IDeg only. However, the ~28% IDeg dose sparing effect of “add‐on” liraglutide with IDegLira versus IDeg alone was paralleled by a ~22% decrease in the dose of liraglutide with IDegLira versus liraglutide alone.^27^ Thus, with the FRC IDegLira, the dose of the GLP‐1RA component, driven by the BI dose, may result in underestimation, especially in obese people who benefit from less BI and more GLP‐1RA.

### 3.2 The studies with iGlaLixi (LixiLan)

The RCTs with the FRC IGla‐100 and lixisenatide (iGlaLixi) are summarized in Table 3.

**TABLE 3:** Randomized controlled trials with the once‐daily FRC iGlarLixi (LixiLan). The values of age, BMI, diabetes duration and HbA1c are presented as means. The outcomes are expressed as the effect of iGlarLixi vs. comparator.

STUDY	Tretament Duration (weeks)	Baseline Treatment	Comparator(s)	*N*	Age (years)	BMI (kg/m^2^)	Diabetes Duration (years)	HbA1c (%)	Outcomes
LixiLan‐PoC^10^	24	Metformin	Gla‐100	323	56	32.1	6.7	8.1	HbA1c ↓ BW ↓ Hypogl. =
LixiLan‐O^37^	30	OGLA^a^	2 separate comparators: Gla‐100 Lixisenatide	1.170	58	31.7	8.8	8.1	HbA1c ↓ BW ↓ Hypogl. =
LixiLan‐L^41^	30	BI+OGLA^a^	IGla‐100	736	60	31.2	12	8.5	HbA1c ↓ BW ↓ Hypogl. =
LixiLan‐G^43^	30	GLP‐1RA + oral agents	GLP‐1RA + oral agents	554	59.5	32.9	11.1	7.9	HbA1c ↓ BW ↑ Hypogl. ↑
Solimix^44^	26	BI+OCLA^a^	Twice daily premixed insulin Asp 30/70	887	59.8	29.9	13	8.6	HbA1c ↓ BW ↓ Hypogl. ↓

^a^ Oral glucose‐lowering agents.

The LixiLan PoC is a proof‐of‐concept study which evaluated the effects of iGlaLixi (with a FRC 2:1, i.e. each 2 Units of IGla‐100 contain 1 μg lixisenatide) versus IGla‐100.^10^ The people studied had a relatively short duration of T2DM, were obese and had a moderate increase in HbA1c on metformin only treatment. At the end of the study, both iGlaLixi and IGla‐100 were highly effective in lowering HbA1c below the target HbA1c of 7.0%, with a small but statistically significant difference in favour of iGlaLixi (6.3% vs. 6.5%, 45 mmol/mol vs. 48 mmol/mol) primarily because of the greater reduction of PPG as indicated by a test meal which, however, was liquid and not solid. IGlaLixi treatment was associated with a small reduction in body weight (−1 kg vs. +0.5 kg of IGla‐100) and no increase in the risk of hypoglycaemia.

The LixiLan‐O trial examined the FRC iGlaLixi versus its separate components IGla‐100 and lixisenatide, respectively, in people with T2DM on OGLA.^37^ IGlaLixi was more effective in reducing HbA1c as compared with each one of the two components. In the lixisenatide‐only arm, FPG was higher as compared with the iGlarLixi arm, but lixisenatide had a robust effect on PPG at breakfast and lunch, although not at dinner, a pattern similar to that of the iGlarLixi arm. The results are consistent with the LixiLan‐O trial conducted in Japanese subjects (with the FRC iGlarLixi 1:1)^38^ and in the Asian‐Pacific (LixiLan‐O‐AP trial, FRC 1:1 or 2:1),^39^ and in the Mexican populations studied.^40^

Lixilan‐L study evaluated iGlaLixi in people with T2DM not at the target HbA1c whilst receiving BI and OGLA treatment.^41^ The people studied had a longer T2DM duration and poorer glycaemic control, suggesting a more advanced stage of diabetes compared with the population of PoC.^10^ After 6 weeks of titration of IGla‐100 leading to a decrease of HbA1c to 8.1% (baseline value), people were randomized to either continuation of titrated IGla‐100 or treatment with iGlarLixi. Two different FRCs were used (2 units IGlar:1 μg Lixi, and 3 units IGlar:1 μg Lixi). At the end of study, HbA1c decreased more with iGlarLixi vs. Gla‐100 (6.9% vs. 7.5%), with 1.5 kg less body weight and no increase of hypoglycaemia. Lixilan‐L study shows the robust effect of iGlarLixi on post‐breakfast PG, but a progressively lower effect on PPG post‐lunch and post‐dinner.^41^ This is consistent with the results of Lixilan‐O^37^ and suggests progressive waning of the effects of the lixisenatide given in the morning at the time of lunch, and especially at dinner. A similar study has recently been conducted in a group of Chinese people with T2DM on BI and OGLA.^42^

The LixiLan‐G study examined the effects of iGlarLixi (with two ratios, 2 Units of iGla:1 μg Lixi, and 3 Units of iGla:1 μg Lixi) in obese people with T2DM not at target HbA1c during treatment with GLP‐1RAs at maximum tolerated doses.^43^ Switching to treatment with iGlarLixi resulted in more effective lowering of HbA1c (from 7.8% to 6.7%) as compared with lixisenatide (from 7.8% to 7.4%). With iGlarLixi, there was an increase in body weight by +1.9 kg with iGlarLixi vs. −1.4 kg with lixisenatide, and an increased risk of hypoglycaemia.

The SoliMix trial examined people with T2DM not at the target on BI and OCLA and randomized to either iGlarLixi or to twice daily premixed insulin (IAsp 30/70).^44^ At the end of the study, HbA1c was lower with iGlarLixi (7.3%) vs. premixed insulin (7.5%), with lower body weight and a reduced risk for hypoglycaemia.

No RCT has prospectively examined the initiation of treatment with the FRC iGlarLixi versus basal‐bolus insulin regimen. However, a retrospective analysis has compared the two treatment regimens using the propensity score matching involving 1628 US adults with T2D not at the target on BI and OCLA with a baseline HbA1c of 9.2% (SoliSymplify Real‐World study).^45^ After 6 months of treatment, the decrease in HbA1c was similar with both treatments. However, the absolute values reached in each group were far from ideal (8.5% and 8.4%, iGlaLixi and basal‐bolus insulin, respectively), indicating inadequate titration of insulin with both treatments.

Similar to the DUAL studies, the iGlarLixi studies have also shown more frequent adverse GI effects with the FRC primarily in the first month of treatment, followed by subsequent disappearance thereafter as compared with OGLA, BI, and pre‐mixed insulin regimens.

#### 3.2.1 Comments to the Lixilan studies

The LixiLan studies mimic the findings of the efficacy and safety as well as the GI side effects of the free combination IGla‐100 and lixisenatide of the GetGoal‐L and GetGoal DUO‐1 studies in comparison with titrated BI Gla‐100.^20^, ^21^

Similar to the DUAL studies, the LixiLan studies also confirmed the well expected benefits of two powerful glucose lowering drugs co‐formulated in a FRC as compared with only one of the two components. Thus, neither the LixiLan studies have shown how much the FRC iGlarLixi approaches the results of the free combination of IGla‐100 and lixisenatide when given separately and independently titrated.^20^, ^21^

Of note, in all studies, iGlaLixi (morning dosing) appeared efficacious in lowering PPG at breakfast, but less so at lunch and even less at dinner, likely because of waning in the evening of the effects of lixisenatide given in the morning.^41^ This confirms that the FRC iGlarLixi has the same limitation of the pharmacodynamics (PD) of lixisenatide given once a day observed in the studies with the free combination lixisenatide and BI.^20^, ^21^

The SoliMix trial indicates the superiority of iGlaLixi to twice a day premixed insulin in people with T2DM previously on BI.^44^ However, advancing therapy to premixed insulin following failure of BI is a suboptimal choice for the generality of people who need treatment intensification.^7^, ^8^ Given the well known limitations of premixed insulin preparations in optimizing glycaemic control,^46^ the poor choice of the premixed insulin regimen as control may indirectly favour the iGlaLixi treatment.

### 3.3 The studies with IcoSema

Recently, three of the four studies of the COMBINE programme with the FRC of the weekly BI icodec and the weekly GLP‐1RA semaglutide (IcoSema) have been published (COMBINE 1, COMBINE 2, and COMBINE 3).^11^, ^12^, ^13^ COMBINE 1 compared the efficacy and safety of IcoSema with icodec in people inadequately controlled on daily BI (Table 4).^11^ At the end of the study, HbA1c decreased from the mean baseline value of 8.2% by −1.55% with IcoSema and −0.89% with icodec, confirming the superiority of the former (*p* < 0.0001). With IcoSema, the risk of hypoglycaemia was lower (superirority confirmed, *p* < 0.0001), and body weight decreased (−3.7 kg) whereas it increased with icodec (+1.89 kg). As expected, more GI adverse events occurred with IcoSema.^11^ In COMBINE 2, people with uncontrolled T2DM and obesity previously on semaglutide with/without additional OGLA were randomized to once weekly semaglutide or IcoSema (Table 4).^12^ As expected, at the end of study, HbA1c decreased more with IcoSema than with semaglutide (to 6.65% vs. 7.1%, respectively). The estimated treatment difference (ETD) was −4.85% (95% CI −6.13, −3.57) mmol/mol (−0.44 [95% CI −0.56, −0.33]%‐points), confirming the superiority of the FRC IcoSema (*p* < 0.0001). There was no difference in the risk of hypoglycaemia between the two regimens. The mean change in body weight from baseline to week 52 was +0,84 kg with IcoSema and −3.7 kg with semaglutide. The proportion of participants reporting GI adverse events (primarily GI) was comparable between the treatment groups (IcoSema 31.4%; semaglutide 34.4%).^12^ COMBINE 3 has examined the efficacy and safety of IcoSema vs. basal‐bolus insulin therapy in people with T2DM inadequately controlled on daily BI (Table 4).^13^ At the end of study, the mean changes of HbA1c with IcoSema and BBT were no different (IcoSema, from mean baseline 8.3%–6.89%; BBT from 8.29% to 6.89%) confirming the non‐inferiority of the FRC versus BBT. With IcoSema, the risk of hypoglycaemia was lower, and the change in body weight confirmed superiority (end of study −3.56 kg with IcoSema vs. +3.16 kg with BBT). Interestingly, there was a large difference in weekly insulin dose (IcoSema 196 U, BBT 466 U). Not surprisingly, more adverse GI events occurred with IcoSema versus BBT.^13^

**TABLE 4:** Randomized controlled trials with the once‐weekly FRC IcoSema (COMBINE). The values of age, BMI, diabetes duration and HbA1c are presented as means. The outcomes are expressed as effect of IcoSema vs. comparator.

STUDY	Treatment Duration (weeks)	Baseline treatment	Comparator(s)	*N*	Age (years)	BMI (kg/m^2^)	Diabetes Duration (years)	HbA1c (%)	Outcomes
COMBINE 1^11^	52	BI±OGLA^a^	Icodec+placebo	1291	61	29.9	15	8.2	HbA1c ↓ BW ↓ Hypogl. ↓
COMBINE 2^12^	52	GLP‐1 RA ± OGLA^a^	Semaglutide	683	8	31.1	13	8.0	HbA1c ↓ BW ↑ Hypogl. =
COMBINE 3^13^	52	BI ± OGLA^a^	Basal‐bolus insulin	679	60	30.4	14	8.3	HbA1c = BW ↓ Hypogl. ↓

^a^ Oral glucose‐lowering agents.

A fourth COMBINE study is ongoing (COMBINE 4) and is expected to terminate by the end of 2025 (NCT06269107 ClinicalTrials.gov). COMBINE 4 compares treatment with IcoSema or IGla‐100 for 40 weeks (followed by 5 weeks of follow‐up) of 474 people with T2DM naïve to insulin and GLP‐1RA. and inadequately controlled on OGLA (HbA1c ≥8.0%). The primary aim is difference in the end of study HbA1c. Seconday aims are differences in body weight and risk for hypoglycaemia.

At present there is no information relating to the possible development of an additional FRC of the weekly BI efsitora alfa^47^ plus the dual GLP‐1‐ Glucose‐dependent Insulinotropic Peptide (GIP) RA tirzepatide.^48^

#### 3.3.1 Comments to the IcoSema studies

The COMBINE studies have tested the FRC IcoSema versus each one of its two components icodec (COMBINE 1),^11^ and the GLP‐1RA semaglutide (COMBINE 2),^12^ respectively. COMBINE 1 and COMBINE 2 confirm with the weekly FRC IcoSema the well expected advantages of treatment with two drugs (BI and GLP‐1RA) as compared with one drug only, as already shown with daily FRCs IDegLira and iGlarLixi.

The COMBINE studies offer a clear example of the insulin dose‐sparing effect of GLP‐1RA given the observation of the ~50% lower insulin dose with IcoSema versus icodec, notably with better glycaemic control in COMBINE 1,^11^ and 57% lower insulin dose with similar glycaemic control versus BBT in COMBINE 3.^13^ Importantly, COMBINE 3 suggests the possibility for people with T2DM already on BBT of deintensification of their complex insulin regimens with daily basal and prandial insulins^49^ switching to a simple weekly dosing of FRC.

Similar to the observation with the daily FRC IDegLira,^27^ also with the weekly FRC in the COMBINE studies^11^, ^12^ the dose of the GLP‐1RA component semaglutide delivered weekly was low (0.48 and 0.54 mg/week, respectively), well below the doses approved and used for treatment of T2DM,^50^ and considerably lower than those proven efficacious in obese people.^51^

Of note, when initiation of IcoSema is compared with icodec (COMBINE 1) or BBT (COMBINE 3), it takes a considerably longer time for IcoSema to control FPG versus the comparators. In COMBINE 1, there was a transient elevation of FPG which then decreased to values not different from icodec only at 18 weeks.^11^ This is similar to COMBINE 3 in which IcoSema resulted in a decrease of HbA1c and self‐monitored pre‐breakfast PG to values no different from those of BBT only at 18 and 24 weeks, respectively.^13^ This lag in glycaemic control of the weekly FRC IcoSema does not occur when the weekly BI icodec is given alone.^24^, ^52^, ^53^, ^54^ This different outcome in COMBINE and ONWARDS studies is likely explained by the lower starting dose of the BI component icodec of the FRC IcoSema in the COMBINE studies (only 40 units/week) as compared with the dose of icodec in ONWARDS studies (70 units/weeks).

## 4 DISCUSSION

All the RCTs of the daily and weekly dosing of fFRCs of BI plus GLP‐1RA have proven efficacy as compared with only one or only the other one, of its two components. No prospective, randomized trial, however, hitherto has compared the FRCs with the free, separate dosing of both its components independently titrated. The PK of IDegLira and iGlaLixi suggest lower s.c. absorption of liraglutide when combined with IDeg^16^ and lixisenatide when combined with IGla‐100,^22^ respectively. This might result in lower plasma availability of the GLP‐1RA after the injection of FRCs as compared with separate dosing of its components. Also, the PK of semaglutide is different when given with IcoSema as compared with semaglutide alone; however, in the opposite direction to liraglutide and lixisenatide.^26^

At present, there are only indirect comparisons and retrospective observations in which either no difference,^55^, ^56^, ^57^, ^58^ or greater efficacy on FPG with a trend for lower HbA1c^59^ has been reported with free versus FRCs. Despite the absence of ad hoc RCTs comparing FRCs with free combinations of its components titrated independently in the largely heterogeneous population with T2DM with quite different needs of BI and GLP‐1RA, one can, however, imagine that FRCs have some beneficial effects in some people. As indicated by the available studies, people on OGLA who need to advance therapy with injectables, or people already on BI only, or on GLP‐1RA only not at target HbA1c will experience a reduction in HbA1c with the FRCs, and will also benefit from a lower body weight and reduced risk of hypoglycaemia (vs. BI only). However, at the same time, it is expected that the daily FRCs will be less successful in people who need higher doses of BI (>60 U/day) and/or in those people who need higher doses of GLP‐1RA for a given dose of BI. The latter is of particular relevance to people with obesity who may benefit from the weekly semaglutide^25^ at doses >0.5 mg/week^50^, ^51^ or the dual agonist tirzepatide.^48^

### 4.1 Differences between the FRCs IDegLira and iGlaLixi

At present there are no prospective RCTs comparing the FRCs IDegLira and iGlarLixi, only indirect comparisons and retrospective observations. There appears to be no difference in lowering HbA1c^60^, ^61^, ^62^ or a marginally greater efficacy of IDegLira along with a greater effect on body weight.^63^ However, the properties of the different components of the two FRCs may explain the different effects observed in people with T2DM.

**FIGURE 1:**
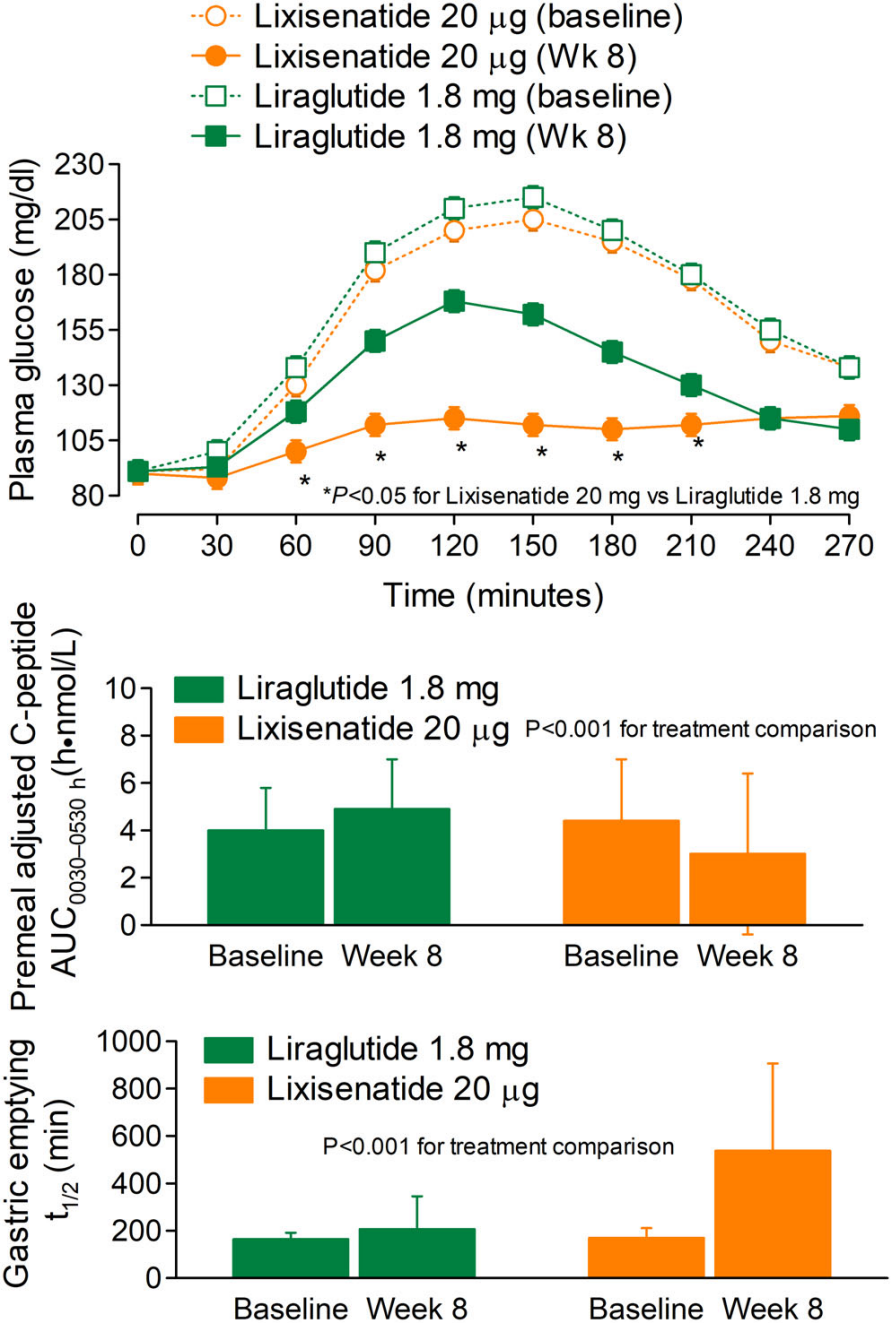
Different mechanisms underlying lowering of PPG with maximal doses of liraglutide and lixisenatide in people with T2DM treated to target with IGla‐100.^18^ *Upper panel*: PG response to a standard solid meal. *Center panel*: C‐peptide responses to a solid standard meal (AUC_0030‐0530_). *Lower panel*: Gastric emptying after a solid standard meal. Adapted and modified from Meier et al.^18^ with permission of the author and the American Diabetes Association.

**FIGURE 2:**
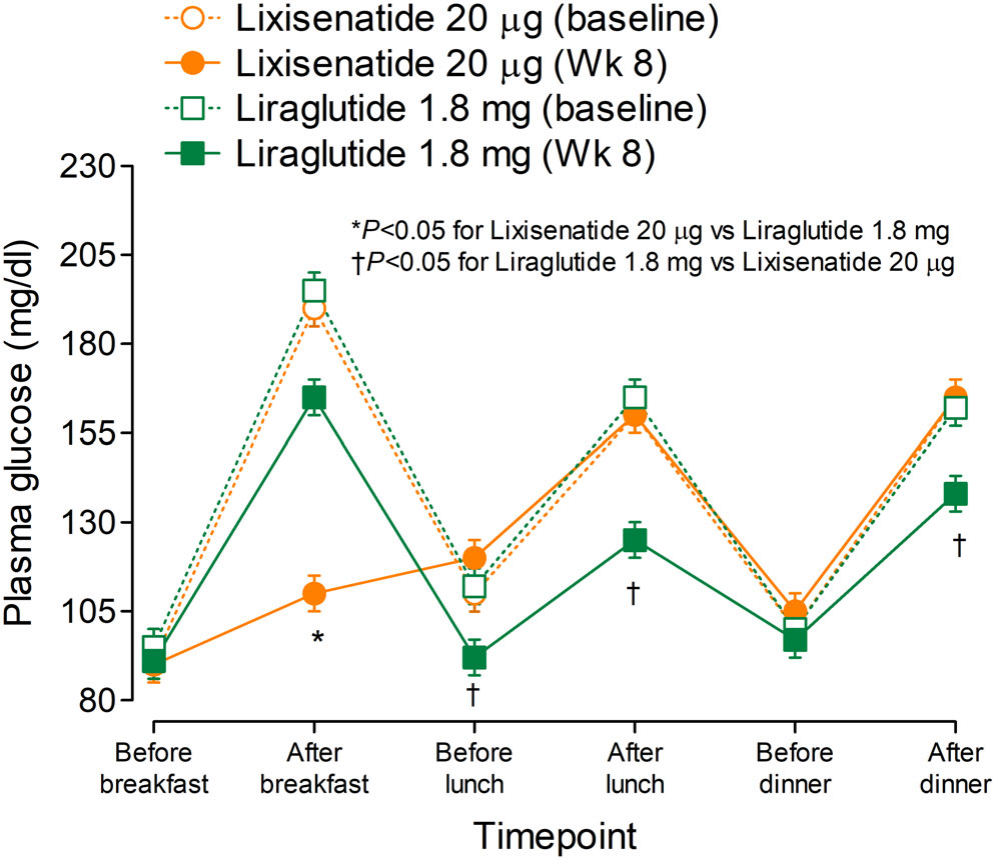
Twenty‐four hour PG profiles after 8 weeks of treatment with maximal doses of liraglutide or lixisenatide in people with T2DM treated to target with BI iGla‐100 as compared with baseline values. Note the greater effect of lixisenatide on PPG at breakfast, and the greater effect of liraglutide on PPG at lunch and dinner when lixisenatide is less effective instead. Adapted and modified from Meier et al.^18^ with permission of the author and the American Diabetes Association.

Regarding the BI components, insulin degludec has a longer duration of action and a flatter pharmacodynamic profile over the 24 h as compared with IGla‐100^64^ and is associated with a lower risk for nocturnal hypoglycaemia.^65^ Regarding the GLP‐1RA components, liraglutide is an acylated long‐acting GLP‐1RA analogue with a half‐life of approximately 13 h^66^ and, therefore, has a prolonged effect over 24 h.^67^ In contrast, lixisenatide is a short‐acting GLP‐1RA analogue with a half‐life of 2.8 h^22^, ^68^ and will exert its effect primarily on the meal taken after dosing or meals taken within 4–6 h after dosing, as shown in the GetGoal‐L clinical trial.^20^ Being a short‐acting GLP‐1RA analogue, lixisenatide lowers the PPG primarily by delaying gastric emptying markedly, although to a highly variable extent, depending on the baseline rate of gastric emptying.^69^ Because of such a mechanism, lixisenatide paradoxically reduces endogenous insulin secretion in response to meal ingestion (Figure 1).^18^ These effects of lixisenatide occur at doses below the usual therapeutic dose of 20 μg.^70^ In contrast, the long‐acting liraglutide primarily stimulates endogenous insulin secretion in response to meal ingestion while still delaying gastric emptying, although to a lesser extent (Figure 1)^18^ because of tachyphylaxis which may occur with long‐acting GLP‐1RAs.^71^ Although the PK/PD of lixisenatide suggests twice daily dosing like exenatide, lixisenatide has been approved for once‐a‐day dosing based unfortunately on only one study in people with T2DM on metformin‐only treatment.^72^ Surprisingly, no studies with twice‐ versus once‐daily lixisenatide have been performed in people with more advanced progression of T2DM requiring injectables. As expected with a short‐acting GLP‐1RA given onceaday, the robust effects of morning dosing of lixisenatide observed at breakfast are to some extent reduced at lunch and nearly wane at the time of the evening meal.^20^ This is quite different from the effects of liraglutide which is less powerful in lowering PPG at breakfast as compared with lixisenatide, but more consistent in controlling PPG at lunch and dinner (Figure 2).^18^, ^31^, ^41^ These differences between lixisenatide and liraglutide observed in free combinations with BI^18^ are understandably carried over in the respective FRCs with BI.^31^, ^41^

There are no head‐to‐head studies comparing the adverse GI effects and tolerability of IDegLira and iGlarLixi. In a network meta‐analysis for the first 12 weeks iGlarLixi was associated with less nausea and vomiting as compared with liraglutide, semaglutide, dulaglutide and exenatide QW.^73^ However, as in the case of most research related to GLP‐1RAs symptoms were assessed using the suboptimal participant ‘self‐report’, rather than a validated measure, and the important distinction between loss of appetite (not bothersome) and nausea (which is) was not made. In future such studies, appropriate assessment of GI symptoms should be planned.

It should also be appreciated that there is no information about differences in the timing of the risk of hypoglycaemia with IDegLira and iGlarLixi. However, considering the different effects on breakfast PPG of lixisenatide as compared with Liraglutide (both given in the morning and associated with BI), with iGlarLixi one should monitor the risk of hypoglycaemia especially in the second half of the morning, whereas with IDegLira the risk might be greater later in the afternoon when insulin resistance is reduced as compared with the morning.^74^

### 4.2 Who would benefit more from the FRC IDegLira, iGlaLixi, or IcoSema?

Based on the RCTs, IDegLira exhibits a similar PPG‐lowering effect at each meal during the day, albeit slightly more at lunch and at the evening meal as compared with breakfast (Figure 1). In contrast, the morning dose of iGlarLixi exerts the greatest effect at breakfast, and less effect at lunch and especially at dinner (Figure 2).^18^, ^31^, ^41^ iGlaLixi improves post‐breakfast PG more than IDegLira^18^ confirming the importance of delaying gastric emptying more than enhancing insulin secretion in lowering PPG.^75^ Therefore, iGlarLixi appears more suitable for people who usually take their largest CHO‐rich meal at breakfast and have only light, low CHO meals later in the day. Alternatively, the iGlaLixi might be given prior to the largest CHO meal of the day, provided that the other meals are poor in CHO. As already mentioned, the short half‐life of lixisenatide strongly suggests a twice‐a‐day use, but studies are needed to test this hypothesis and establish the degree of potential tachyphylaxis.^76^

The more recent weekly FRC IcoSema^11^ may prove useful in those people who are on the weekly BI icodec already, or are candidates for it, and need to improve PPG. However, given the longer time required with IcoSema to control hyperglycaemia versus daily BI and BBT,^11^, ^13^ care should be taken in people with remarkably poor control to improve hyperglycaemia initially with either daily BI or BBT prior to the switch to IcoSema. Alternatively, more aggressive titration algorithms than those used in COMBINE 1^11^ and COMBINE 3^13^ should be adopted, although this might translate into a too rapid an increase in the weekly semaglutide dose and side GI effects.

### 4.3 Lessons of the FRC studies

The FRCs make it practically easier and more convenient to administer BI and GLP‐1RA as one dosing with higher chances of better compliance and adherence versus free combinations of the individual components.^77^ However, while the replacement of the BI component appears appropriate with the daily FRCs IdegLira and iGlarLixi, that of the GLP‐1RA component may be less optimal. This limitation may apply especially to iGlarLixi in which the GLP‐1RA component has a much shorter half‐life as compared with that of IDegLira. However, even with IDegLira, the dose of liraglutide may be suboptimal, and the bioavailability reduced with the FRC.^16^ Also, with the most recent weekly FRC IcoSema,^11^, ^12^, ^13^ the dose of the GLP‐1RA component semaglutide may be underestimated for the needs of some individuals, especially when obese.

One interesting application of IDegLira has recently been reported in a retrospective study demonstrating that this FRC can be used in the deintensification of the complex basal‐bolus insulin regimen in older people with T2DM.^78^ The potential for maintaining or even improving glycaemic control with lower risk of hypoglycaemia, and only one injection as compared with multiple injections a day would be an important advantage of simplification for the old, frail population.^49^ These results are similar to those of previous studies based on free combinations of BIs and GLP‐1RAs in people previously on a basal‐bolus insulin regimen, which have established the feasibility of reducing the use of mealtime insulin.^79^, ^80^ The recent COMBINE 3 study^13^ suggesting that deintensification of BBT might be possible also with the weekly FRC IcoSema calls for an ad hoc trial in this regard.

## 5 CONCLUSIONS

The availability of FRCs of basal insulin (BI) and GLP‐1RAs offers a simplified option to advance therapy in T2DM to further improve glycaemic control, lower body weight without increasing, even lowering, the risk of hypoglycaemia. The combination of BI and GLP‐1RA also reduces, or may avoid, the need for rapid‐acting insulin at meal‐time,^7^, ^8^ even in people already on a basal‐bolus insulin regimen,^78^, ^79^, ^80^ and is non‐inferior to BBT.^13^

Interestingly, when the FRCs became available some 10 years ago, the predominant recommendation to advance therapy beyond oral hypoglycaemic agents (OGLA) was to begin with the introduction of BI.^81^ Therefore, the introduction of FRCs was a significant innovation, offering the advantages of combining BI and GLP‐1RA in a convenient and simplified manner. However, the more recent introduction of long‐acting GLP‐1RAs and the once‐weekly dual agonist tirzepatide (GLP plus GLP‐1) has changed the therapeutic options, making it now possible to intensify treatment of the GLP‐1RA component relative to BI, as compared with the FRCs.

Given the considerable heterogeneity among people with T2DM and their varying therapeutic needs, the FRCs remain to have an important role today, for example, as the first injectable when advancing therapy is required after failure of OGLA. However, frequent longitudinal monitoring of glycaemic control remains essential to determine whether the person with T2DM should continue on FRC therapy or switch to separate dosing and titration of BI and GLP‐1RA to enrich treatment with BI and/or GLP‐1RA as needed.

## AUTHOR CONTRIBUTIONS

G.B.B. wrote the first draft. All other authors have equally contributed to rewriting and editing the text. The final text was approved by all. G.B.B is the guarantor of this work, has full access to the new data in the review, and takes responsibility for the integrity of the analysis thereof and its accuracy.

## FUNDING INFORMATION

This article was funded in part by the University of Perugia and a private donation (F.D.), and did not receive any additional funding from the public, commercial, or not‐for‐profit sectors. G.P. is supported by MUR ‐PRIN, 2022KZ4KMY.

## CONFLICT OF INTEREST STATEMENT

G.B.B. has received honoraria for lecturing from Sanofi. F.P. receives clinical trial funding, advisory board, and lecture fees from Abbott, AstraZeneca, Lilly, Novo Nordisk, Sanofi. P.L. and C.G.F. have no conflicts of interest. G.P. has received honoraria for lecturing and advisory boards for Amgen, Bruno Farmaceutici, EG, AstraZeneca, Bayer, Boehringer Ingelheim, Daiichi Sankyo, Echosens, Lilly, Menarini Diag, Merck, Novartis, Novo Nordisk, Pfizer, PikDare, Roche Diag, Sanofi, Servier; clinical trial funding from Novartis, Novo Nordisk, Merck. M.H. and D.R.O. have received lecture fees from Bayer, Boehringer Ingelheim, Roche, and Sanofi.

## PEER REVIEW

The peer review history for this article is available at https://www.webofscience.com/api/gateway/wos/peer-review/10.1111/dom.16616.

## Data Availability

Not relevant to review article.
